# Risk Factors for the Development of Colistin Resistance during Colistin Treatment of Carbapenem-Resistant Klebsiella pneumoniae Infections

**DOI:** 10.1128/spectrum.00381-22

**Published:** 2022-06-02

**Authors:** Po-Han Huang, Wen-Yin Chen, Sheng-Hua Chou, Fu-Der Wang, Yi-Tsung Lin

**Affiliations:** a Division of Infectious Diseases, Department of Medicine, Taipei Veterans General Hospital, Taipei, Taiwan; b Division of Infectious Diseases, Department of Paediatrics, Taipei Veterans General Hospital, Taipei, Taiwan; c Institute of Emergency and Critical Care Medicine, National Yang Ming Chiao Tung University, Taipei, Taiwan; Keck School of Medicine of the University of Southern California; Addis Ababa University; ICMR-National Institute of Cholera and Enteric Diseases; University of Medicine and Pharmacy at Ho Chi Minh City

**Keywords:** colistin, resistance emergence, protective factors, carbapenem, *Klebsiella pneumoniae*

## Abstract

Colistin is one of the last-resort options for carbapenem-resistant Klebsiella pneumoniae (CRKP) infections if novel antibiotics are unavailable, where the development of colistin resistance during treatment represents a major challenge for clinicians. We aimed to investigate the risk factors associated with the development of colistin resistance in patients with CRKP infections following colistin treatment. We conducted a retrospective case-control study of patients with CRKP strains available before and after colistin treatment at a medical center in Taiwan, between October 2016 and November 2020. Cases (*n* = 35) included patients with an initial colistin-susceptible CRKP (ColS-CRKP) strain and a subsequent colistin-resistant CRKP (ColR-CRKP) strain. Controls (*n* = 18) included patients with ColS-CRKP as both the initial and subsequent strains. The 30-day mortality rate after the subsequent CRKP isolation was not different between cases and controls (12/35 [34%] versus 5/18 [28%] [*P* = 0.631]). *bla*_KPC_ (*n* = 38) and *bla*_OXA-48_ (*n* = 11) accounted for the major mechanisms of carbapenem resistance. Alterations in *mgrB* were found in 18/35 (51%) ColR-CRKP strains, and *mcr-1* was not detected in any of the strains. More patients received combination therapy in the control group than in the case group (17/18 versus 21/35 [*P* = 0.008]). The logistic regression model indicated that combination therapy with tigecycline was protective against the acquisition of colistin resistance (odds ratio, 0.17; 95% confidence interval, 0.05 to 0.62 [*P *= 0.008]). We observed that the inclusion of tigecycline in colistin treatment mitigated the risk of acquiring colistin resistance. These results offer insight into using the combination of tigecycline and colistin for the treatment of CRKP infections in antimicrobial stewardship.

**IMPORTANCE** Treatment of carbapenem-resistant Klebsiella pneumoniae (CRKP) infections is challenging due to the limited options of antibiotics. Colistin is one of the last-resort antibiotics if novel antimicrobial agents are not available. It is crucial to identify modifiable clinical factors associated with the emergence of resistance during colistin treatment. Here, we found that the addition of tigecycline to colistin treatment prevented the acquisition of colistin resistance. Colistin-tigecycline combination therapy is therefore considered a hopeful option in antimicrobial stewardship to treat CRKP infections.

## INTRODUCTION

Infection by carbapenem-resistant Klebsiella pneumoniae (CRKP) causes significant morbidity and mortality, and its global spread poses a major threat to public health (1). Colistin remains an important “last-resort” antibiotic for patients with CRKP infections because novel agents against CRKP are unavailable in many countries (2). However, colistin resistance may develop from the addition of cationic groups (phosphoethanolamine or 4-amino-4-deoxy-l-arabinose) to lipid A within lipopolysaccharides. Modifications of lipid A are usually mediated by chromosomal mutations of genes encoding the two-component systems PmrAB and PhoPQ, by the inactivation of the *mgrB* gene, or from the plasmid-mediated *mcr-1* gene (3–6). The development of colistin resistance in CRKP makes treatment even more challenging and leads to an increased mortality rate (7, 8).

Several studies have explored the risk factors for colistin resistance during CRKP infections (9–11). Previously, we have shown that colistin exposure is an independent risk factor for the *in vivo* emergence of colistin resistance in CRKP (12). However, studies regarding the risk factors for colistin resistance development after colistin treatment for CRKP infections are lacking. Previous studies have shown conflicting results with regard to the use of combination therapy with colistin, where combination therapy may either suppress the emergence of colistin-resistant K. pneumoniae subpopulations or not reduce the emergence of colistin resistance in carbapenem-resistant Gram-negative bacteria (13, 14). However, the above-mentioned studies included a limited number of CRKP isolates.

We conducted this case-control study to identify potential risk factors for colistin resistance development following colistin treatment for CRKP infections as well as to clarify the impacts of different antibiotic combinations on resistance emergence.

## RESULTS

### Study population and clinical characteristics.

During the 49-month study period, we identified 35 (case) and 18 (control) patients from whom a colistin-resistant CRKP (ColR-CRKP) strain and a colistin-susceptible CRKP (ColS-CRKP) strain were isolated, respectively, 7 to 28 days after intravenous colistin treatment for a ColS-CRKP infection. There was no outbreak of CRKP infection identified at our hospital, and there were no more than two patients in the same unit infected by CRKP during the same period. The colistin MICs for the initial and subsequent CRKP strains are shown in Table S1 in the supplemental material. The demographic and clinical characteristics of the case and control groups are shown in Table 1. This study found a high mortality rate for patients with CRKP infections. There were no significant differences between the case and control groups in the in-hospital mortality rate (54% [19/35] versus 44% [8/18] [*P* = 0.497]), 30-day mortality rate after the initial CRKP isolation (23% [8/35] versus 22% [4/18] [*P* = 1.000]), or 30-day mortality rate after the subsequent CRKP isolation (34% [12/35] versus 28% [5/18] [*P* = 0.631]).

**TABLE 1:** Demographic and clinical characteristics of case and control groups^*g*^

Variable	Value for group		*P* value
	Case (*n* = 35)	Control (*n* = 18)	
Mean age (yrs) (±SD)	65 (±17)	66 (±15)	0.833
No. (%) of male patients	21 (60)	15 (83)	0.085
Median LOS before initial CRKP isolation (days) (IQR)	27 (12–45)	13 (1–28)	0.035
Mean no. of days between initial and subsequent CRKP isolation (±SD)	17 (±6)	17 (±5)	0.650
Median Charlson comorbidity index (IQR)	8 (5–10)	8 (5–9)	0.698
No. (%) of patients with comorbidity			
Malignancy	18 (51)	7 (39)	0.386
Solid tumor	15 (43)	6 (33)	0.502
Hematological malignancy	3 (9)	1 (6)	1.000
Diabetes mellitus	18 (51)	8 (44)	0.630
Chronic kidney disease^*a*^	23 (66)	11 (61)	0.741
ESRD^*b*^	11 (31)	2 (11)	0.177
Heart failure	8 (23)	3 (17)	0.730
Coronary artery disease	11 (31)	3 (17)	0.333
PAOD	7 (20)	2 (11)	0.701
Cirrhosis	7 (20)	2 (11)	0.701
Cerebrovascular accident	9 (26)	7 (39)	0.322
COPD	3 (9)	4 (22)	0.211
Connective tissue disease^*c*^	4 (11)	0 (0)	0.287
Solid-organ transplantation	6 (17)	2 (11)	0.701
Chronic ventilator dependence^*d*^	7 (20)	3 (17)	1.000
No. (%) of patients with immunosuppression^*e*^	29 (83)	10 (56)	0.049
Corticosteroid treatment	25 (71)	9 (50)	0.123
Immunosuppressant treatment	7 (20)	3 (17)	1.000
Chemotherapy	0 (0)	1 (6)	0.340
Neutropenia	2 (6)	2 (11)	0.598
Mean APACHE II score (±SD)	21 (±6)	20 (±10)	0.710
No. (%) of patients with medical device			
Mechanical ventilator	28 (80)	15 (83)	1.000
Tracheostomy	7 (20)	6 (33)	0.326
Central venous catheter	29 (81)	15 (83)	1.000
Urinary catheter	28 (80)	14 (78)	1.000
Nasogastric or nasojejunal tube	32 (91)	17 (94)	1.000
Surgical drain^*f*^	14 (40)	6 (33)	0.635
Surgery	5 (14)	6 (33)	0.154
No. (%) of patients with no source control	1 (3)	0 (0)	1.000
Median length of ICU stay (IQR)	11 (3–17)	12 (1–15)	0.539
In-hospital mortality rate [no. (%) of patients]	19 (54)	8 (44)	0.497
30-day mortality rate after 1st CRKP isolation [no. (%) of patients]	8 (23)	4 (22)	1.000
30-day mortality rate after 2nd CRKP isolation [no. (%) of patients]	12 (34)	5 (28)	0.631

a Defined according to KDIGO 2012 clinical practice guidelines for the evaluation and management of chronic kidney disease (31).

b Defined as an estimated glomerular filtration rate (GFR) of <15 mL/min/1.73 m^2^ or dialysis dependence for more than 30 days.

c Defined according to the Charlson comorbidity index.

d Defined as ventilator dependence for more than 30 days prior to the initial CRKP isolation.

e Defined as meeting one of the following criteria: use of corticosteroids or immunosuppressants, receiving chemotherapy, or neutropenia. Use of corticosteroids was defined as receipt of a corticosteroid at a dose of ≥10 mg per day of prednisolone for more than 5 days in the 30 days prior to the subsequent CRKP isolation. Chemotherapy was defined as the receipt of cytotoxic antineoplastic drugs for cancer within 30 days before the subsequent CRKP isolation. Neutropenia was defined as a peripheral absolute neutrophil count of <0.5 × 10^9^ cells/L.

f Defined as the presence of drainage tubes after surgeries or invasive procedures.

g CRKP, carbapenem-resistant Klebsiella pneumoniae; IQR, interquartile range; LOS, length of hospital stay; ESRD, end-stage renal disease; PAOD, peripheral arterial occlusive disease; COPD, chronic obstructive pulmonary disease; APACHE, Acute Physiology and Chronic Health Evaluation; ICU, intensive care unit.

The most common CRKP infections among the 53 patients included pneumonia (*n* = 27; 51%), primary bacteremia (*n* = 8; 15%), and urinary tract infection (*n* = 5; 9%). The culture sites of the initial and subsequent CRKP strains are shown in Table S2. The median numbers of days of colistin treatment for cases and controls were 9 days (interquartile range [IQR], 7 to 14 days) and 12 days (IQR, 10 to 13 days), respectively (*P* = 0.118) (Table S3). Twenty-one (60%) case and 17 (94%) control patients received combination therapy (*P* = 0.008). The regimens used in combination therapy are shown in Table 2. The most common combination was colistin plus tigecycline, which was used in 13 (37%) and 14 (78%) patients in the case and control groups, respectively (*P* = 0.005). Ceftazidime-avibactam, meropenem-vaborbactam, and imipenem-cilastatin-relebactam were unavailable at the hospital during the study period.

**TABLE 2:** Combination therapy during the interval between the initial and subsequent carbapenem-resistant K. pneumoniae strains

Antibiotic therapy	No. (%) of patients receiving therapy in group		*P* value
	Case (*n* = 35)	Control (*n* = 18)	
Combination therapy	21 (60)	17 (94)	0.008
Colistin + tigecycline	13 (37)	14 (78)	0.005
Colistin + carbapenem^*a*^	11 (31)	9 (50)	0.187
Colistin + aminoglycoside^*b*^	2 (6)	0 (0)	0.543
Colistin + tigecycline + carbapenem^*a*^	4 (11)	4 (22)	0.421
Colistin + aminoglycoside^*b*^ + carbapenem^*a*^	1 (3)	0 (0)	1.000

a Includes ertapenem, imipenem-cilastatin, meropenem, and doripenem.

b Includes amikacin and gentamicin.

### Microbiological characteristics of CRKP strains.

Table 3 shows the antimicrobial susceptibilities of the 53 initial CRKP isolates. Twenty-six (84%) case and 14 (78%) control CRKP isolates were susceptible to tigecycline, with no statistical difference being observed (*P* = 0.478). Overall, carbapenemase-producing strains were the majority in both the case and control groups (*n* = 52; 98%) (Table S4). The most common carbapenemase gene was *bla*_KPC_ (*n* = 38; 72%), followed by *bla*_OXA-48_ (*n* = 11; 21%), *bla*_NDM_ (*n* = 2; 4%), and *bla*_IMP_ (*n* = 1; 2%). K47 was the most common capsular type in the CRKP strains (*n* = 37; 70%), followed by K64 (*n* = 8; 15%) and KN2 (*n* = 4; 8%) (Table S4). No statistical difference was observed between cases and controls in the proportions of carbapenemases and capsular types of the initial CRKP strains.

**TABLE 3:** Antimicrobial susceptibilities of 53 initial CRKP strains

Antibiotic(s)	No. (%) of patients with susceptible initial strain in group		*P* value
	Case (*n* = 35)	Control (*n *= 18)	
Ceftazidime	0 (0)	0 (0)	
Cefepime	1 (3)	1 (6)	1.000
Piperacillin-tazobactam	0 (0)	0 (0)	
Ertapenem	0 (0)	0 (0)	
Imipenem	0 (0)	0 (0)	
Amikacin	34 (97)	16 (89)	0.263
Gentamicin	19 (54)	8 (44)	0.497
Ciprofloxacin	0 (0)	1 (6)	0.340
Levofloxacin	0 (0)	1 (6)	0.340
Tigecycline	29 (83)	13 (72)	0.478
Trimethoprim-sulfamethoxazole	6 (17)	5 (28)	0.478

The quantification of *pmrH* mRNA expression showed that 97% (34/35) of the ColR-CRKP strains had higher expression levels than their colistin-susceptible counterparts (Table S1). Only TVGH-CR22 had no significant difference in *pmrH* mRNA expression levels between the ColS-CRKP and ColR-CRKP strains. We quantified another gene of the *pmrHFIJKLM* operon, *pmrK*, in strain TVGH-CR22, and the *pmrK* mRNA expression level of the ColR-CRKP strain was higher than that of its colistin-susceptible counterpart (Table S1). No ColR-CRKP strain carrying the *mcr-1* gene was detected. Alterations in *mgrB* were found in 18 (51%) ColR-CRKP strains, while amino acid substitutions in PmrAB and PhoPQ were detected in 14 (40%) and 2 (6%) ColR-CRKP strains. Among the sequential CRKP isolates of the case group, 30 (86%) initial ColS-CRKP strains had pulsed-field gel electrophoresis (PFGE) patterns (≤3 different bands) similar to those of their subsequent ColR-CRKP counterparts. The PFGE results are shown in Fig. S1.

### Risk factors for the emergence of colistin resistance.

As shown in Tables 1 and 2, patients in the case group were more likely to be male and immunosuppressed. Patients in the case group had also stayed in the hospital for a longer duration before the initial ColS-CRKP isolation, and fewer of them received colistin-tigecycline combination therapy for CRKP infections. Age, sex, and variables with *P *values of <0.1 in the univariate analysis were entered into a stepwise backward selection logistic regression model (Table 4). No use of colistin-tigecycline combination therapy was the only significant risk factor for the development of colistin resistance. Colistin-tigecycline combination therapy was found to be protective against colistin resistance (odds ratio, 0.17; 95% confidence interval, 0.05 to 0.62 [*P* = 0.008]) (Table 4).

**TABLE 4:** Univariate and multivariate analyses of clinical factors associated with the development of colistin resistance in CRKP strains during colistin treatment^*a*^

Variable	Univariate analysis		Multivariate analysis	
	OR (95% CI)	*P* value	OR (95% CI)	*P* value
Age		0.833		
Male sex	0.30 (0.07–1.23)	0.085		
LOS before initial CRKP isolation		0.035		
Immunosuppression	3.87 (1.08–13.90)	0.049		
Colistin-tigecycline combination therapy	0.35 (0.14–0.87)	0.005	0.17 (0.05–0.62)	0.008

a CRKP, carbapenem-resistant Klebsiella pneumoniae; OR, odds ratio; CI, confidence interval; LOS, length of hospital stay.

## DISCUSSION

In the current study, the inclusion of tigecycline with colistin treatment was found to protect against the development of colistin resistance in patients with CRKP infections treated with colistin. We also found a notably high mortality rate among patients with CRKP strains. Our previous work demonstrated that colistin treatment is an independent risk factor for the *in vivo* emergence of colistin resistance in CRKP (12). Therefore, it is crucial to identify modifiable clinical factors associated with the development of resistance during colistin treatment. The use of combination antibiotic therapy has been proposed as a potential method to mitigate this development. An *in vitro* study conducted by Deris et al. found that colistin-doripenem combination therapy reduced the emergence of colistin-resistant multidrug-resistant K. pneumoniae strains, but only one CRKP isolate was included in this study (13). Clinical studies on the risk factors for the development of colistin-resistant strains during colistin treatment for CRKP infections are limited. Recently, Dickstein et al. conducted a secondary analysis of a randomized controlled trial (RCT) and concluded that colistin-carbapenem combination therapy could not prevent colistin resistance compared to colistin monotherapy in patients infected with carbapenem-resistant organisms; however, only five initial colistin-susceptible isolates were CRKP (14). In line with this secondary analysis, our study did not find a protective effect of colistin-carbapenem combination therapy, but our study found that the inclusion of tigecycline with colistin treatment prevented the development of colistin resistance. While the availability and uptake of novel antimicrobial agents are critical elements in the fight against CRKP, the findings of our study indicate that colistin-tigecycline combination therapy for CRKP infections may be a promising option in antimicrobial stewardship.

In the secondary analysis conducted by Dickstein et al. (14), the authors aimed to compare colistin resistance development following colistin-meropenem combination therapy versus colistin monotherapy in patients infected with carbapenem-resistant organisms. These organisms included several types of carbapenem-resistant Gram-negative bacteria, including *Enterobacterales* (such as Escherichia coli and K. pneumoniae) and nonfermenting Gram-negative bacilli (such as Acinetobacter baumannii and Pseudomonas aeruginosa). The study design was robust, as no other antimicrobials targeting Gram-negative bacteria were allowed, and the follow-up isolates in this RCT were regularly obtained through surveillance rectal swabs. Our research was an observational study and gave us the opportunity to compare different antibiotic regimens. With a median duration (10 days) of colistin treatment similar to that reported by Dickstein et al. (14), our real-world observation data corresponded to the notion that the addition of carbapenem to colistin therapy could not prevent resistance development. Our findings also indicate that further RCTs are warranted to validate the role of tigecycline in preventing colistin resistance during colistin treatment of CRKP.

The combination of tigecycline and colistin in the treatment of CRKP infections has been well studied *in vitro* and *in vivo*. Using a time-kill assay, Pournaras et al. found that the colistin-tigecycline combination is synergistic and bactericidal against K. pneumoniae carbapenemase (KPC)-producing CRKP isolates (15). Within a mouse model, Fergadaki et al. suggested that treatment with tigecycline, either as monotherapy or in combination with other antibiotics (including colistin and/or meropenem), significantly prolonged survival in KPC-producing CRKP infections (16). Using a time-kill assay, Tian et al. found that the combination of polymyxin B and tigecycline demonstrated bactericidal activity against CRKP strains with heteroresistance to polymyxin B and tigecycline (17). Clinical studies on the effect of colistin-tigecycline combination therapy on CRKP infections are usually conducted before the availability of novel agents against this organism, where better results are reported with regimens containing colistin and tigecycline (18, 19). Our study was not designed to evaluate the impact of combination therapy on patient outcomes but does indicate a plausible benefit of colistin-tigecycline combination therapy. This potential benefit is particularly desirable in real-world practice, as the absence of routine laboratory testing for colistin resistance prevents physicians from detecting the emergence of resistance in a timely manner.

This study had several limitations. We had a relatively small sample size, and the statistical analysis may not have sufficient power to extrapolate the results to the overall population. However, studies on the collection of paired sequential CRKP isolates to evaluate the development of colistin resistance are limited in the literature. In addition, only patients with CRKP isolates available before and after colistin treatment were included, and the collection of specimen cultures was decided by the treating physician. Patients with CRKP infections treated successfully with colistin did not have subsequent cultures available and were not enrolled as our study controls. Therefore, the population at risk for colistin resistance may be overrepresented. Furthermore, no ColR-CRKP strain carrying the *mcr-1* gene was detected in our study, so whether the findings could be generalizable to ColR-CRKP strains with *mcr-1* acquisition was unknown. Finally, we did not perform whole-genome sequencing for phylogenetic analysis. While the acquisition of colistin resistance in this study was not limited to *in vivo* emergence, and some cases might acquire colistin-resistant strains exogenously, the result is more reflective of real-world practice.

In conclusion, our case-control study demonstrated that combination therapy with tigecycline protects against the development of colistin resistance in patients with CRKP infections receiving colistin treatment. Further prospective interventional studies are needed to validate this association as well as to examine whether this protective effect can be translated into a higher likelihood of clinical success. Our results also offer insight into antimicrobial stewardship, and colistin-tigecycline combination therapy may be a promising strategy to curb colistin resistance when treating CRKP infections.

## MATERIALS AND METHODS

### Study design and patients.

This study was conducted at the Taipei Veterans General Hospital in Taiwan between October 2016 and November 2020. A case-control study was undertaken to identify the risk or protective factors associated with the development of colistin resistance following colistin treatment of CRKP infections. We defined cases as patients from whom an initial ColS-CRKP strain was isolated and, after 7 to 28 days, a ColR-CRKP strain was isolated. We defined controls as patients from whom a ColS-CRKP strain was isolated initially and after 7 to 28 days of treatment. Both case and control groups experienced ≥5 days of intravenous colistin treatment for the initial ColS-CRKP infections. All patients in each group met the study’s inclusion criteria. Intravenous colistin was administered at a loading dose of 5 mg/kg of body weight followed by a maintenance dose of 2.5 mg × (1.5 × creatinine clearance + 30) every 12 h. The Institutional Review Board of Taipei Veterans General Hospital approved and waived the informed consent of this study.

### Data collection and definitions.

We collected demographic and clinical data from the electronic chart review, including length of stay before the initial ColS-CRKP isolation, comorbidities, Charlson comorbidity index, immunosuppression (through corticosteroid usage, immunosuppressant usage, receipt of chemotherapy, or neutropenia), and Acute Physiology and Chronic and Prevention Evaluation (APACHE) II score calculated 24 h before or after culture collection. The use of mechanical ventilation, the presence of indwelling medical devices, the length of intensive care unit stay, and antibiotics administered during the interval between the initial and subsequent CRKP strain isolations were also included. Combination therapy was defined as the administration of colistin and other antibiotics commonly used to treat CRKP for >72 h, regardless of the MIC or site of infection. These antibiotics included tigecycline, carbapenem, or aminoglycosides. Source control was defined as the removal of infected medical devices or drainage of infected fluid collections within 7 days after obtaining the initial culture.

### Microbiological study of K. pneumoniae strains.

Klebsiella pneumoniae was identified using matrix-assisted laser desorption ionization–time of flight mass spectrometry (bioMérieux). We determined the MICs of antibiotics other than colistin and tigecycline with the Vitek-2 system (bioMérieux), and the results were interpreted according to Clinical and Laboratory Standards Institute criteria (20). In this study, CRKP was defined as a K. pneumoniae isolate that was nonsusceptible (MIC ≥ 2 mg/L) to imipenem or meropenem. Colistin resistance was defined as an MIC of >2 mg/L according to European Committee on Antimicrobial Susceptibility Testing (EUCAST) 2022 version 12.0 guidelines (http://www.eucast.org/clinical_breakpoints), as determined by broth microdilution. Tigecycline susceptibility was based on U.S. Food and Drug Administration criteria (susceptible, MIC ≤ 2 mg/L; resistant, MIC ≥ 8 mg/L), as determined by an Etest (bioMérieux) (21).

As previously described, we used *wzi* sequencing to determine the capsular types of the CRKP strains (22, 23). All CRKP isolates were screened for carbapenemase genes, including *bla*_KPC_, *bla*_OXA-48_, *bla*_IMP_, and *bla*_NDM_, as previously described (24). To determine the colistin resistance mechanism, we identified the presence of the *mcr-1* gene and alterations in the *mgrB*, *phoPQ*, *pmrAB*, and *crrAB* genes as previously described (4, 25). These genes were then amplified by PCR, and the nucleotides were determined by Sanger sequencing. Subsequently, we compared the gene sequences in ColR-CRKP strains to those in their ColS-CRKP counterparts. We then compared the amino acid alignments with Clustal Omega and identified the insertion sequences using ISfinder as previously described (12, 26, 27). Moreover, we evaluated the mRNA expression levels of the *pmrH*, *pmrK*, and 16S rRNA genes using real-time quantitative reverse transcription-PCR as previously described (3, 4, 12). The relative expression of target genes in CRKP strains was compared to that in a colistin-susceptible strain, NTUH-K2044 (expression = 1; colistin MIC = 1 mg/L). The ΔΔ*C_T_* method was used with normalization to 16S rRNA levels for analysis. Primers for PCR are listed in Table S5 in the supplemental material.

To evaluate the genetic relatedness of the initial colistin-susceptible and subsequent colistin-resistant strains in the case group, we conducted PFGE as previously described (28, 29). The results were interpreted according to Tenover criteria, with paired strains being considered genetically indistinguishable or closely related if they had no more than three band differences (30).

### Statistical analyses.

Categorical variables were compared using the chi-square test or Fisher’s exact test, as appropriate. Continuous variables were analyzed using Student’s *t* test or a Mann-Whitney U test, as appropriate. Multivariate analysis was run for age, sex, and all variables with *P *values of <0.1 in univariate analyses using a stepwise backward selection logistic regression model. All statistical analyses were performed using Statistical Package for the Social Sciences software version 23.0 (SPSS, Chicago, IL, USA). For all analyses, we considered a two-tailed *P *value of <0.05 to be statistically significant.

## Supplementary Material

Reviewer comments

## References

[1] Agyeman AA, Bergen PJ, Rao GG, Nation RL, Landersdorfer CB. 2020. A systematic review and meta-analysis of treatment outcomes following antibiotic therapy among patients with carbapenem-resistant *Klebsiella pneumoniae* infections. Int J Antimicrob Agents 55:105833. doi:10.1016/j.ijantimicag.2019.10.014. 31730892

[2] Tamma PD, Aitken SL, Bonomo RA, Mathers AJ, van Duin D, Clancy CJ. 2021. Infectious Diseases Society of America guidance on the treatment of extended-spectrum beta-lactamase producing Enterobacterales (ESBL-E), carbapenem-resistant Enterobacterales (CRE), and *Pseudomonas aeruginosa* with difficult-to-treat resistance (DTR-*P. aeruginosa*). Clin Infect Dis 72:e169–e183. doi:10.1093/cid/ciaa1478. 33106864

[3] Cheng YH, Lin TL, Lin YT, Wang JT. 2016. Amino acid substitutions of CrrB responsible for resistance to colistin through CrrC in *Klebsiella pneumoniae*. Antimicrob Agents Chemother 60:3709–3716. doi:10.1128/AAC.00009-16. 27067316 PMC4879426

[4] Cheng YH, Lin TL, Pan YJ, Wang YP, Lin YT, Wang JT. 2015. Colistin resistance mechanisms in *Klebsiella pneumoniae* strains from Taiwan. Antimicrob Agents Chemother 59:2909–2913. doi:10.1128/AAC.04763-14. 25691646 PMC4394772

[5] Poirel L, Jayol A, Nordmann P. 2017. Polymyxins: antibacterial activity, susceptibility testing, and resistance mechanisms encoded by plasmids or chromosomes. Clin Microbiol Rev 30:557–596. doi:10.1128/CMR.00064-16. 28275006 PMC5355641

[6] Cheng YH, Lin TL, Lin YT, Wang JT. 2018. A putative RND-type efflux pump, H239_3064, contributes to colistin resistance through CrrB in *Klebsiella pneumoniae*. J Antimicrob Chemother 73:1509–1516. doi:10.1093/jac/dky054. 29506266 PMC5961088

[7] Rojas LJ, Salim M, Cober E, Richter SS, Perez F, Salata RA, Kalayjian RC, Watkins RR, Marshall S, Rudin SD, Domitrovic TN, Hujer AM, Hujer KM, Doi Y, Kaye KS, Evans S, Fowler VG, Jr, Bonomo RA, van Duin D, Antibacterial Resistance Leadership Group. 2017. Colistin resistance in carbapenem-resistant *Klebsiella pneumoniae*: laboratory detection and impact on mortality. Clin Infect Dis 64:711–718. doi:10.1093/cid/ciw805. 27940944 PMC5850634

[8] Capone A, Giannella M, Fortini D, Giordano A, Meledandri M, Ballardini M, Venditti M, Bordi E, Capozzi D, Balice MP, Tarasi A, Parisi G, Lappa A, Carattoli A, Petrosillo N. 2013. High rate of colistin resistance among patients with carbapenem-resistant *Klebsiella pneumoniae* infection accounts for an excess of mortality. Clin Microbiol Infect 19:E23–E30. doi:10.1111/1469-0691.12070. 23137235

[9] Richter SE, Miller L, Uslan DZ, Bell D, Watson K, Humphries R, McKinnell JA. 2018. Risk factors for colistin resistance among Gram-negative rods and *Klebsiella pneumoniae* isolates. J Clin Microbiol 56:e00149-18. doi:10.1128/JCM.00149-18. 29976595 PMC6113453

[10] Kontopidou F, Plachouras D, Papadomichelakis E, Koukos G, Galani I, Poulakou G, Dimopoulos G, Antoniadou A, Armaganidis A, Giamarellou H. 2011. Colonization and infection by colistin-resistant Gram-negative bacteria in a cohort of critically ill patients. Clin Microbiol Infect 17:E9–E11. doi:10.1111/j.1469-0691.2011.03649.x. 21939468

[11] Giacobbe DR, Del Bono V, Trecarichi EM, De Rosa FG, Giannella M, Bassetti M, Bartoloni A, Losito AR, Corcione S, Bartoletti M, Mantengoli E, Saffioti C, Pagani N, Tedeschi S, Spanu T, Rossolini GM, Marchese A, Ambretti S, Cauda R, Viale P, Viscoli C, Tumbarello M, ISGRI-SITA (Italian Study Group on Resistant Infections of the Società Italiana Terapia Antinfettiva). 2015. Risk factors for bloodstream infections due to colistin-resistant KPC-producing *Klebsiella pneumoniae*: results from a multicenter case-control-control study. Clin Microbiol Infect 21:1106.e1–1106.e8. doi:10.1016/j.cmi.2015.08.001. 26278669

[12] Huang PH, Cheng YH, Chen WY, Juan CH, Chou SH, Wang JT, Chuang C, Wang FD, Lin YT. 2021. Risk factors and mechanisms of *in vivo* emergence of colistin resistance in carbapenem-resistant *Klebsiella pneumoniae*. Int J Antimicrob Agents 57:106342. doi:10.1016/j.ijantimicag.2021.106342. 33864932

[13] Deris ZZ, Yu HH, Davis K, Soon RL, Jacob J, Ku CK, Poudyal A, Bergen PJ, Tsuji BT, Bulitta JB, Forrest A, Paterson DL, Velkov T, Li J, Nation RL. 2012. The combination of colistin and doripenem is synergistic against *Klebsiella pneumoniae* at multiple inocula and suppresses colistin resistance in an *in vitro* pharmacokinetic/pharmacodynamic model. Antimicrob Agents Chemother 56:5103–5112. doi:10.1128/AAC.01064-12. 22802247 PMC3457376

[14] Dickstein Y, Lellouche J, Schwartz D, Nutman A, Rakovitsky N, Dishon Benattar Y, Altunin S, Bernardo M, Iossa D, Durante-Mangoni E, Antoniadou A, Skiada A, Deliolanis I, Daikos GL, Daitch V, Yahav D, Leibovici L, Rognås V, Friberg LE, Mouton JW, Paul M, Carmeli Y, AIDA Study Group. 2020. Colistin resistance development following colistin-meropenem combination therapy versus colistin monotherapy in patients with infections caused by carbapenem-resistant organisms. Clin Infect Dis 71:2599–2607. doi:10.1093/cid/ciz1146. 31758195

[15] Pournaras S, Vrioni G, Neou E, Dendrinos J, Dimitroulia E, Poulou A, Tsakris A. 2011. Activity of tigecycline alone and in combination with colistin and meropenem against *Klebsiella pneumoniae* carbapenemase (KPC)-producing Enterobacteriaceae strains by time-kill assay. Int J Antimicrob Agents 37:244–247. doi:10.1016/j.ijantimicag.2010.10.031. 21236643

[16] Fergadaki S, Renieris G, Machairas N, Sabracos L, Droggiti DI, Misiakos E, Giamarellos-Bourboulis EJ. 2021. Efficacy of tigecycline alone or in combination for experimental infections by KPC carbapenemase-producing *Klebsiella pneumoniae*. Int J Antimicrob Agents 58:106384. doi:10.1016/j.ijantimicag.2021.106384. 34161789

[17] Tian Y, Zhang Q, Wen L, Chen J. 2021. Combined effect of polymyxin B and tigecycline to overcome heteroresistance in carbapenem-resistant *Klebsiella pneumoniae*. Microbiol Spectr 9:e00152-21. doi:10.1128/Spectrum.00152-21. 34704782 PMC8549724

[18] Tumbarello M, Viale P, Viscoli C, Trecarichi EM, Tumietto F, Marchese A, Spanu T, Ambretti S, Ginocchio F, Cristini F, Losito AR, Tedeschi S, Cauda R, Bassetti M. 2012. Predictors of mortality in bloodstream infections caused by *Klebsiella pneumoniae* carbapenemase-producing *K. pneumoniae*: importance of combination therapy. Clin Infect Dis 55:943–950. doi:10.1093/cid/cis588. 22752516

[19] Qureshi ZA, Paterson DL, Potoski BA, Kilayko MC, Sandovsky G, Sordillo E, Polsky B, Adams-Haduch JM, Doi Y. 2012. Treatment outcome of bacteremia due to KPC-producing *Klebsiella pneumoniae*: superiority of combination antimicrobial regimens. Antimicrob Agents Chemother 56:2108–2113. doi:10.1128/AAC.06268-11. 22252816 PMC3318350

[20] Clinical and Laboratory Standards Institute. 2021. Performance standards for antimicrobial susceptibility testing, 31st ed. Clinical and Laboratory Standards Institute, Wayne, PA.

[21] Wyeth Pharmaceuticals Inc. 2010. Full prescribing information for Tygacil. Wyeth Pharmaceuticals Inc, Philadelphia, PA. https://www.accessdata.fda.gov/drugsatfda_docs/label/2010/021821s021lbl.pdf. Accessed 5 January 2022.

[22] Brisse S, Passet V, Haugaard AB, Babosan A, Kassis-Chikhani N, Struve C, Decré D. 2013. *wzi* gene sequencing, a rapid method for determination of capsular type for *Klebsiella* strains. J Clin Microbiol 51:4073–4078. doi:10.1128/JCM.01924-13. 24088853 PMC3838100

[23] Hsu CR, Lin TL, Pan YJ, Hsieh PF, Wang JT. 2013. Isolation of a bacteriophage specific for a new capsular type of *Klebsiella pneumoniae* and characterization of its polysaccharide depolymerase. PLoS One 8:e70092. doi:10.1371/journal.pone.0070092. 23936379 PMC3732264

[24] Lin Y-T, Su C-F, Chuang C, Lin J-C, Lu P-L, Huang C-T, Wang J-T, Chuang Y-C, Siu LK, Fung C-P. 2019. Appropriate treatment for bloodstream infections due to carbapenem-resistant *Klebsiella pneumoniae* and *Escherichia coli*: a nationwide multicenter study in Taiwan. Open Forum Infect Dis 6:ofy336. doi:10.1093/ofid/ofy336. 30740468 PMC6362312

[25] Liu Y-Y, Wang Y, Walsh TR, Yi L-X, Zhang R, Spencer J, Doi Y, Tian G, Dong B, Huang X, Yu L-F, Gu D, Ren H, Chen X, Lv L, He D, Zhou H, Liang Z, Liu J-H, Shen J. 2016. Emergence of plasmid-mediated colistin resistance mechanism MCR-1 in animals and human beings in China: a microbiological and molecular biological study. Lancet Infect Dis 16:161–168. doi:10.1016/S1473-3099(15)00424-7. 26603172

[26] Madeira F, Park YM, Lee J, Buso N, Gur T, Madhusoodanan N, Basutkar P, Tivey ARN, Potter SC, Finn RD, Lopez R. 2019. The EMBL-EBI search and sequence analysis tools APIs in 2019. Nucleic Acids Res 47:W636–W641. doi:10.1093/nar/gkz268. 30976793 PMC6602479

[27] Siguier P, Perochon J, Lestrade L, Mahillon J, Chandler M. 2006. ISfinder: the reference centre for bacterial insertion sequences. Nucleic Acids Res 34:D32–D36. doi:10.1093/nar/gkj014. 16381877 PMC1347377

[28] Lin YT, Cheng YH, Chuang C, Chou SH, Liu WH, Huang CH, Yang TC, Kreiswirth BN, Chen L. 2020. Molecular and clinical characterization of multidrug-resistant and hypervirulent *Klebsiella pneumoniae* strains from liver abscess in Taiwan. Antimicrob Agents Chemother 64:e00174-20. doi:10.1128/AAC.00174-20. 32152079 PMC7179640

[29] Huang YH, Chou SH, Liang SW, Ni CE, Lin YT, Huang YW, Yang TC. 2018. Emergence of an XDR and carbapenemase-producing hypervirulent *Klebsiella pneumoniae* strain in Taiwan. J Antimicrob Chemother 73:2039–2046. doi:10.1093/jac/dky164. 29800340

[30] Tenover FC, Arbeit RD, Goering RV, Mickelsen PA, Murray BE, Persing DH, Swaminathan B. 1995. Interpreting chromosomal DNA restriction patterns produced by pulsed-field gel electrophoresis criteria for bacterial strain typing. J Clin Microbiol 33:2233–2239. doi:10.1128/jcm.33.9.2233-2239.1995. 7494007 PMC228385

[31] Levin A, Stevens PE, Bilous RW, Coresh J, De Francisco ALM, De Jong PE, Griffith KE, Hemmelgarn BR, Iseki K, Lamb EJ, Levey AS, Riella MC, Shlipak MG, Wang H, White CT, Winearls CG. 2013. Kidney Disease: Improving global outcomes (KDIGO) CKD work group. KDIGO 2012 clinical practice guideline for the evaluation and management of chronic kidney disease. Kidney Int Suppl 3:1–150.

