# Supplementary material for: Risk Factors for the Development of Colistin Resistance during Colistin Treatment of Carbapenem-Resistant Klebsiella pneumoniae Infections
Source: Microbiol Spectr. 2022 Jun 2;10(3):e00381-22. doi: 10.1128/spectrum.00381-22 (PMC9241908; doi:10.1128/spectrum.00381-22)
Supplement: Supplemental file 1 — Tables S1 to S5; Fig. S1. Download spectrum.00381-22-s0001.pdf, PDF file, 0.6 MB [file spectrum.00381-22-s0001.pdf]

Supplementary Table S1. Colistin MIC and mechanisms of colistin resistance in 35 ColR-CRKP strains

Strains	Colistin MIC	*pmrH* mRNA expressions	*pmrK* mRNA expressions	Genetic alternations and amino acid substitutions of ColR-CRKP strains					
				**MgrB**	**PhoP**	**PhoQ**	**PmrA**	**PmrB**	**CrrA**
TVGH-CR1	0.5 vs. ≥64	2.6 ± 0.7 vs. 51.7 ± 6.4						Asp150Tyr	
TVGH-CR2	0.25 vs. ≥64	2.3 ± 1.2 vs. 33.1 ± 28.6						Thr157Pro	
TVGH-CR3	0.25 vs. 32	8.4 ± 8.8 vs. 114.7 ± 69.8						Ser203Pro	
TVGH-CR4	0.5 vs. 32	2.1 ± 2.7 vs. 19.6 ± 8.7						Ser203Pro	
TVGH-CR5	0.5 vs. ≥64	0.9 ± 0.41 vs. 4.13 ± 3.44						Thr157Pro	
TVGH-CR6	0.5 vs. ≥64	1.8 ± 1.9 vs. 13.5 ± 16.8		ND[b]					
TVGH-CR7	0.5 vs. 32	1.3 ± 1.6 vs. 5.8 ± 6.7						Thr157Pro	
TVGH-CR8	0.25 vs. ≥64	2.1 ± 2.5 vs. 15.9 ± 19.4		Gln30Pro				Thr140Pro	
TVGH-CR9	0.5 vs. 32	1.3 ± 1.5 vs. 11.1 ± 14.0		IS*Kpn14*[a]					
TVGH-CR10	0.5 vs. ≥64	4.0 ± 5.5 vs. 13.2 ± 16.3		IS*1R*[a]				Ala246Thr	ND[c]
TVGH-CR11	0.25 vs. ≥64	5.2 ± 7.2 vs.		IS*Kpn26*[a]					

		33.8 ± 46.0					
TVGH-CR12	0.5 vs. 16	4.0 ± 5.6 vs.<br>7.6 ± 10.3		Thr21Asn			
TVGH-CR13	0.5 vs. ≥64	1.5 ± 1.5 vs.<br>13.1 ± 16.0					ND[c]
TVGH-CR14	0.25 vs. ≥64	3.6 ± 5.0 vs.<br>25.8 ± 32.9		IS*903B*[a]			
TVGH-CR15	0.25 vs. ≥64	3.3 ± 4.2<br>29.8 ± 38.4			Gly53Val		
TVGH-CR16	0.5 vs. ≥64	3.0 ± 4.1<br>22.7 ± 29.4		IS*Kpn26*[a]			
TVGH-CR17	0.25 vs. ≥64	1.5 ± 1.6 vs.<br>16.4 ± 21.4			Gly53Ser		
TVGH-CR18	1 vs. ≥64	0.6 ± 0.2 vs.<br>19.0 ± 11.3		Frameshift[d]			
TVGH-CR19	1 vs. 32	0.6 ± 0.2 vs.<br>24.0 ± 16.3		IS*1R*[a]	Glu191Val		
TVGH-CR20	0.5 vs. ≥64	1.0 ± 0.9 vs.<br>10.0 ± 7.3		Deletion[e]			
TVGH-CR21	0.125 vs. 64	0.6 ± 0.5 vs.<br>14.9 ± 10.5		Trp47Cys			
TVGH-CR22	0.5 vs. 32	1.15 ± 0.75 vs.<br>1.37 ± 1.01	3.6 ± 3.0 vs.<br>18.2 ± 11.2				
TVGH-CR23	0.25 vs. ≥64	0.4 ± 0.2<br>21.6 ± 10.5				Leu82Arg	

TVGH-CR24	0.25 vs. ≥64	0.6 ± 0.4<br>12.0 ± 7.6				
TVGH-CR25	0.25 vs. ≥64	0.6 ± 0.4 vs.<br>24.8 ± 17.0	Frameshift[f]			
TVGH-CR26	1 vs. ≥64	0.4 ± 0.3<br>21.1 ± 13.6		Lys46Glu		
TVGH-CR27	1 vs. 32	1.6 ± 0.4 vs.<br>19.6 ± 7.5	ND[b]			
TVGH-CR28	<0.125 vs. ≥64	0.3 ± 0.1 vs.<br>27.8 ± 15.1		Leu96Pro		
TVGH-CR29	<0.125 vs. 32	0.3 ± 0.5 vs.<br>6.5 ± 4.7			Thr157Pro	
TVGH-CR30	<0.125 vs. ≥64	0.7 ± 0.3 vs.<br>13.9 ± 3	IS*903B*[a]			
TVGH-CR31	<0.125 vs. 64	1.7 ± 0.5<br>37.2 ± 3.2	ND[b]			
TVGH-CR32	0.25 vs. ≥64	3.5 ± 3.3<br>22.6 ± 24.4	IS*Kpn26*[a]			
TVGH-CR33	0.5 vs. 64	1.2 ± 0.4<br>25.5 ± 6.7	IS*1R*[a]			
TVGH-CR34	0.5 vs. 64	0.8 ± 0.4<br>15.6 ± 9.7				IS*5*[g]
TVGH-CR35	0.25 vs. ≥64	0.6 ± 0.1<br>26.7 ± 5.8			Leu245Arg	

Abbreviations: MIC, minimum inhibitory concentration; ColR-CRKP, colistin-resistant carbapenem-resistant *Klebsiella pneumoniae*; ND, non-detectable

a. Insertion sequences were located at nucleotide positions 18, 46, 70, 61, 71, 123, 95, 80, and 117 in *mgrB* coding region of TVGH-CR9, -CR10, -CR11, -CR14, -CR16, -CR19, -CR30, -CR32, and -CR33 ColR-CRKP strains, respectively.
b. The *mgrB* gene was present in the colistin-susceptible CRKP strain, but not detected in its paired ColR-CRKP strain.
c. The *crrA* gene was present in the colistin-susceptible CRKP strain, but not detected in its paired ColR-CRKP strain.
d. The adenine and cytosine at nucleotide positions 118 and 119 in *mgrB* coding region of ColR-CRKP strain was absent, resulting in *mgrB* frameshift.
e. The DNA fragment showed a 12-bp deletion of *mgrB*, resulting in a 4-amino acid deletion (residues 8-11).
f. The adenine at nucleotide position 9 in *mgrB* coding region of ColR-CRKP strain was absent, resulting in *mgrB* frameshift.
g. Insertion sequence, IS*5*, was located at the *crrA*-*crrC* intergenic region (the nucleotide position 65 in *crrC* promoter region) of TVGH-CR34 ColR-CRKP strain.

Supplementary Table S2. Culture sites of the initial and subsequent CRKP strains

Culture sites	Case (n = 35)	Control (n = 18)
**Initial strain**		
Blood	11 (31)	5 (28)
Respiratory system	18 (51)	6 (33)
Urine	3 (9)	1 (6)
Abscess	2 (6)	0 (0)
Tip	1 (3)	0 (0)
Wound	0 (0)	4 (22)
Ascites	0 (0)	1 (6)
Bile	0 (0)	1 (6)
**Subsequent strain**		
Blood	4 (11)	4 (22)
Respiratory system	24 (69)	7 (39)
Urine	2 (6)	0 (0)
Wound	2 (6)	4 (22)
Ascites	1 (3)	2 (11)
Pleural fluid	1 (3)	0 (0)
Abscess	1 (3)	0 (0)
Bile	0 (0)	1 (6)

Data are expressed as No. (%) unless otherwise specified.

Abbreviation: CRKP, carbapenem-resistant *Klebsiella pneumoniae*

Supplementary Table S3. Length of antibiotic use during the interval between the initial and subsequent CRKP strains

Antibiotics	Case (n = 35)	Control (n = 18)	*P* value
Intravenous colistin	9 (7–13)	12 (10–13)	.118
Inhaled colistin	0 (0–10)	0 (0–6)	.139
1st and 2nd generation cephalosporin[a]	0 (0–0)	0 (0–0)	1.000
3rd and 4th generation cephalosporin[b]	1 (0–5)	0 (0–4)	.170
Penicillin[c]	0 (0–3)	0 (0–2)	.586
Carbapenem[d]	2 (0–9)	7 (1–11)	.261
Fluoroquinolone[e]	0 (0–3)	0 (0–1)	.490
Aminoglycoside[f]	0 (0–0)	0 (0–0)	.351
Tigecycline	2 (0–7)	9 (4–13)	.004
Vancomycin	0 (0–0)	0 (0–0)	.309
Teicoplanin	0 (0–0)	0 (0–3)	.389
Glycopeptide[g]	0 (0–3)	0 (0–3)	.908
Linezolid	0 (0–0)	0 (0–0)	.652
Daptomycin	0 (0–0)	0 (0–0)	.205
Anti–MRSA antibiotics[h]	1 (0–5)	0 (0–3)	.473
Sulbactam[i]	0 (0–2)	0 (0–0)	.338
Azithromycin	0 (0–0)	0 (0–0)	.499
Clindamycin	0 (0–0)	0 (0–0)	.473
Metronidazole	0 (0–0)	0 (0–0)	.743

Data are expressed as the median days (IQR) of patients receiving the listed antibiotics during the interval between initial and subsequent CRKP isolation.

Abbreviations: CRKP, carbapenem-resistant *Klebsiella pneumoniae*; MRSA, methicillin-resistant *Staphylococcus aureus*

a. Includes cefazolin and cefuroxime.
b. Includes ceftriaxone, ceftazidime, cefepime, and cefixime.
c. Includes amoxicillin-clavulanate, ampicillin-sulbactam, and piperacillin-tazobactam.
d. Includes ertapenem, imipenem-cilastatin, meropenem, and doripenem.
e. Includes ciprofloxacin, levofloxacin, and moxifloxacin.
f. Includes amikacin and gentamicin.
g. Includes vancomycin and teicoplanin.
h. Includes vancomycin, teicoplanin, linezolid, and daptomycin.
i. Includes sulbactam and ampicillin-sulbactam.

Supplementary Table S4. Carbapenemases and capsular types of the initial CRKP strains

Variable	Case (n = 35)	Control (n = 18)	*P* value
**Carbapenemases**			
$bla_{\text{KPC-2}}$	23 (66)	15 (83)	0.177
$bla_{\text{OXA-48}}$	9 (26)	2 (11)	0.296
bla_{IMP}	0 (0)	1 (6)	0.340
bla_{NDM}	2 (6)	0 (0)	0.543
**Capsular types**			
K47	22 (63)	15 (83)	0.124
K64	7 (20)	1 (6)	0.240
KN2	4 (11)	0 (0)	0.287

Data are expressed as No. (%) unless otherwise specified.

Abbreviation: CRKP, carbapenem-resistant *Klebsiella pneumoniae*

Supplementary Table S5. PCR primer sequences

Target	Primer name	Sequence (5'-3')	Purpose	Reference
K47	K47wzyF	CAATACTGGAGCTGCAAAGG	Amplification	Huang YH *et al*., 2018 (1)
	K47wzyR	TCCAACATTAAACGCACCAC	Amplification	Huang YH *et al*., 2018 (1)
K64	K64wzyF	CTTTTAGGGCTACGGCACC	Amplification	Pan YJ *et al*., 2015 (2)
	K64wzyR	CCGCGCGCAGGAACATTAG	Amplification	Pan YJ *et al*., 2015 (2)
wzi	K-wzi_F	GTGCCGCGAGCGCTTTCTATCTTGGTATTCC	Amplification and sequencing	Brisse *et al*., 2013 (3)
	K-wzi_R	GAGAGCCACTGGTTCCAGAACTTCACCGCTTCACCGC	Amplification	Brisse *et al*., 2013 (3)
KPC	KPC-F3	GTTGATGTCACTGTATCGC	Amplification	Huang YH *et al*., 2018 (1)
	KPC-R	TTACTGCCCGTTGACGCCCAATCC	Amplification	Monteiro *et al*., 2012 (4)
OXA	OXA48-F2	GGGACGTTATGCGTGTAT	Amplification	Huang PH *et al*., 2021 (5)
	OXA-48R	TCGAGCATCAGCATTTTGTC	Amplification	Ma L *et al*., 2015 (6)
IMP	IMP-F	GGAATAGAGTGGCTTAATTCTC	Amplification	Ellington *et al*., 2007 (7)
	IMP-R	CCAAACCACTACGTTATC	Amplification	Ellington *et al*., 2007 (7)
NDM	NDM-F	CTATTTACTAGGCCTCGCATT	Amplification	Pasteran *et al*., 2012 (8)
	NDM-R	ATAAAACGCCTCTGTCACAT	Amplification	Pasteran *et al*., 2012 (8)
mcr-1	mcr-1-F2	GTTCTTGTGGCGAGTGTTG	Amplification	Cheng YH *et al*., 2021 (9)
	mcr-1-R2	GATCGGATTGACATAGCTACG	Amplification	Cheng YH *et al*., 2021 (9)
pmrH	pmrH-RT-F	CCGCATCCGTAGCCTGAA	qRT-PCR	Cheng YH *et al*., 2015 (10)
	pmrH-RT-R	CGTGGGTCTGGCGATCAT	qRT-PCR	Cheng YH *et al*., 2015 (10)
mgrB	mgrB-F	CACCACCTCAAAGAGAAGGCGTTC	Amplification and sequencing	Cannatelli *et al*., 2013 (11)
	mgrB-R	AACACGTTTTGAAACAAGTCGATGATTC	Amplification	Cannatelli *et al*., 2013 (11)
pmrD	pmrD-F	CTTGCGGCCTTGATATTCATG	Amplification and sequencing	Cheng YH *et al*., 2015 (10)

	pmrD-R	CCAGTGTCGAAATAGATGCTTC	Amplification	Cheng YH *et al*., 2015 (10)
pmrAB	pmr5-F2	GCGTCTATTTGCATGGTCTG	Amplification and sequencing	Huang PH *et al*., 2021 (5)
	pmrB-R	TCTTATCGTCCTGCTTGCCA	Amplification and sequencing	Huang PH *et al*., 2021 (5)
	pmr5-F	GATAATCTGTTCTCCAC	Amplification and sequencing	Cheng YH *et al*., 2015 (10)
	pmr3-R2	CGACACTATCTTCAACCTGA	Amplification and sequencing	Huang PH *et al*., 2021 (5)
	pmrb5-com-F	AAATCGCGGATCCGCAC	Sequencing	Cheng YH *et al*., 2015 (10)
phoPQ	pho5-F	TCGAGGGTCATATCCTG	Amplification	Cheng YH *et al*., 2015 (10)
	KP2135-seqR1	GTCGGGCCAGTTAAGCGTTA	Amplification and sequencing	Cheng YH *et al*., 2015 (10)
	pho5-F2	TTCCATGCAGTATGCGC	Sequencing	Huang PH *et al*., 2021 (5)
	phoP-seqF1	CAAAGTGGTCAGCAAAGATTC	Sequencing	Cheng YH *et al*., 2015 (10)
	phoQ-deF1	CGGTGACCCTTATCTAC	Sequencing	Cheng YH *et al*., 2015 (10)
crrAB	TupA-F	AAGTCCCAAAAGAGGCAAAC	Amplification and sequencing	Cheng YH *et al*., 2016 (12)
	H236-2575-R	GTGAGGCCATCAAATTCTCG	Amplification and sequencing	Cheng YH *et al*., 2016 (12)
	CrrAB-SeqF1	CTCCCACGTAAGATAGTCAC	Sequencing	Cheng YH *et al*., 2016 (12)

References

1. Huang YH, Chou SH, Liang SW, Ni CE, Lin YT, Huang YW, Yang TC. 2018. Emergence of an XDR and carbapenemase-producing hypervirulent *Klebsiella pneumoniae* strain in Taiwan. J Antimicrob Chemother 73:2039-2046.
2. Pan YJ, Lin TL, Lin YT, Su PA, Chen CT, Hsieh PF, Hsu CR, Chen CC, Hsieh TC, Wang JT. 2015. Identification of capsular types in carbapenem-resistant *Klebsiella pneumoniae* strains by wzc sequencing and implications for capsule depolymerase treatment. Antimicrobial Agents and Chemotherapy 59:1038–1047.
3. Brisse S, Passet V, Haugaard AB, Babosan A, Kassis-Chikhani N, Struve C, Decré D. 2013. *Wzi* Gene sequencing, a rapid method for determination of capsular type for *Klebsiella* strains. J Clin Microbiol 51:4073-4078.
4. Monteiro J, Santos AF, Asensi MD, Peirano G, Gales AC. 2009. First report of KPC-2-producing *Klebsiella pneumoniae* strains in Brazil. Antimicrob Agents Chemother 53:333-334.

5. Huang PH, Cheng YH, Chen WY, Juan CH, Chou SH, Wang JT, Chuang C, Wang FD, Lin YT. 2021. Risk factors and mechanisms of *in vivo* emergence of colistin resistance in carbapenem-resistant *Klebsiella pneumoniae*. Int J Antimicrob Agents 57:106342.
6. Ma L, Wang JT, Wu TL, Siu LK, Chuang YC, Lin JC, Lu MC, Lu PL. 2015. Emergence of OXA-48-Producing *Klebsiella pneumoniae* in Taiwan. PLoS One 10:e0139152.
7. Ellington MJ, Kistler J, Livermore DM, Woodford N. 2007. Multiplex PCR for rapid detection of genes encoding acquired metallo-beta-lactamases. J Antimicrob Chemother 59:321-322.
8. Pasteran F, Albornoz E, Faccone D, Gomez S, Valenzuela C, Morales M, Estrada P, Valenzuela L, Matheu J, Guerriero L, Arbizú E, Calderón Y, Ramon-Pardo P, Corso A. 2012. Emergence of NDM-1-producing *Klebsiella pneumoniae* in Guatemala. J Antimicrob Chemother 67:1795-1797.
9. Cheng YH, Chou SH, Huang PH, Yang TC, Juan YF, Kreiswirth BN, Lin YT, Chen L. 2021. Characterization of a mcr-1 and CRISPR-Cas System Co-harboring Plasmid in a Carbapenemase-Producing High-Risk ST11 *Klebsiella pneumoniae* Strain. Front Microbiol 12:762947.
10. Cheng YH, Lin TL, Pan YJ, Wang YP, Lin YT, Wang JT. 2015. Colistin resistance mechanisms in *Klebsiella pneumoniae* strains from Taiwan. Antimicrob Agents Chemother 59:2909-2913.
11. Cannatelli A, D'Andrea MM, Giani T, Pilato VD, Arena F, Ambretti S, Gaibani P, Rossolini GM. 2013. *In vivo* emergence of colistin resistance in *Klebsiella pneumoniae* producing KPC-type carbapenemases mediated by insertional inactivation of the PhoQ/PhoP *mgrB* regulator. Antimicrob Agents Chemother 57:5521-5526.
12. Cheng YH, Lin TL, Lin YT, Wang JT. 2016. Amino Acid Substitutions of CrrB Responsible for Resistance to Colistin through CrrC in *Klebsiella pneumoniae*. Antimicrob Agents Chemother 60:3709-3716.

Supplementary Figure F1

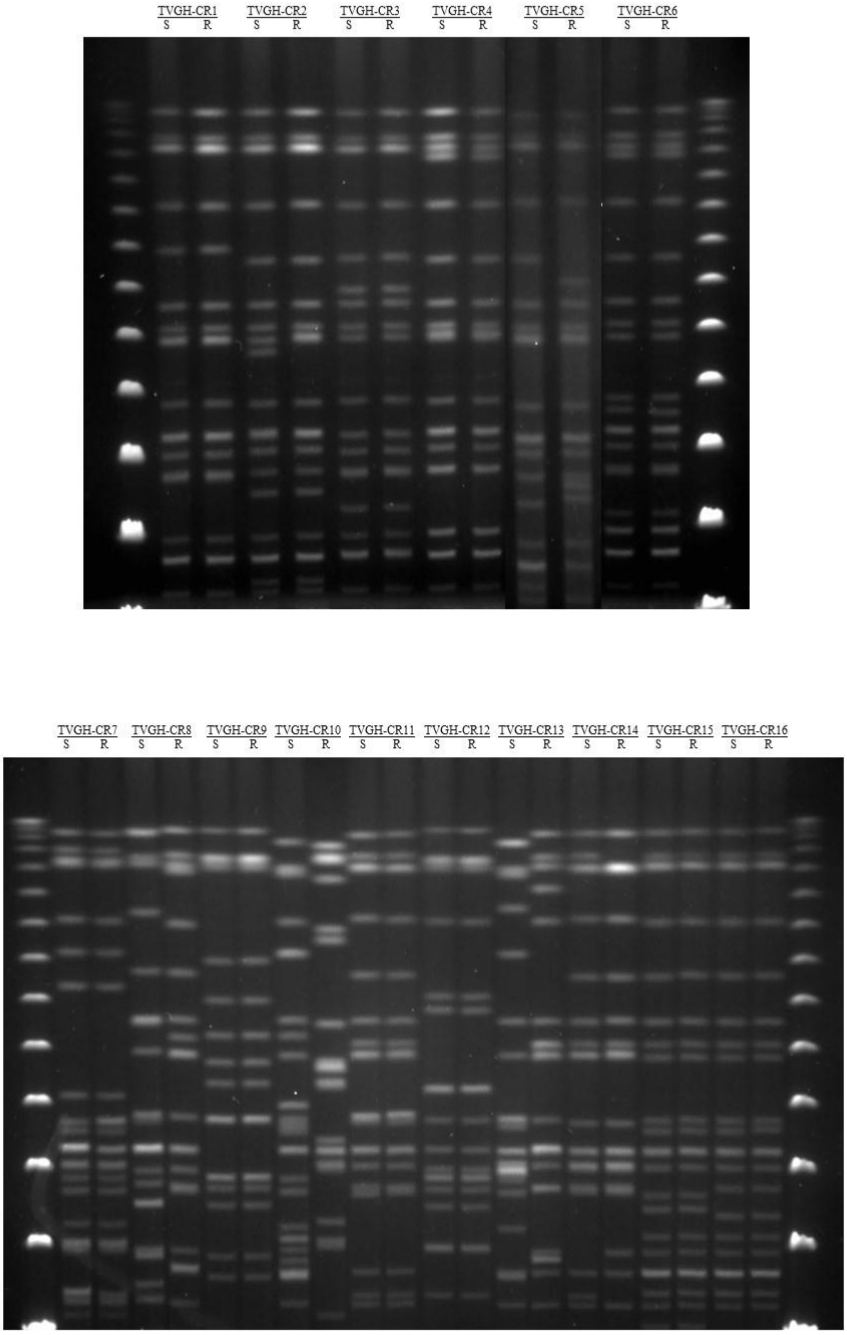

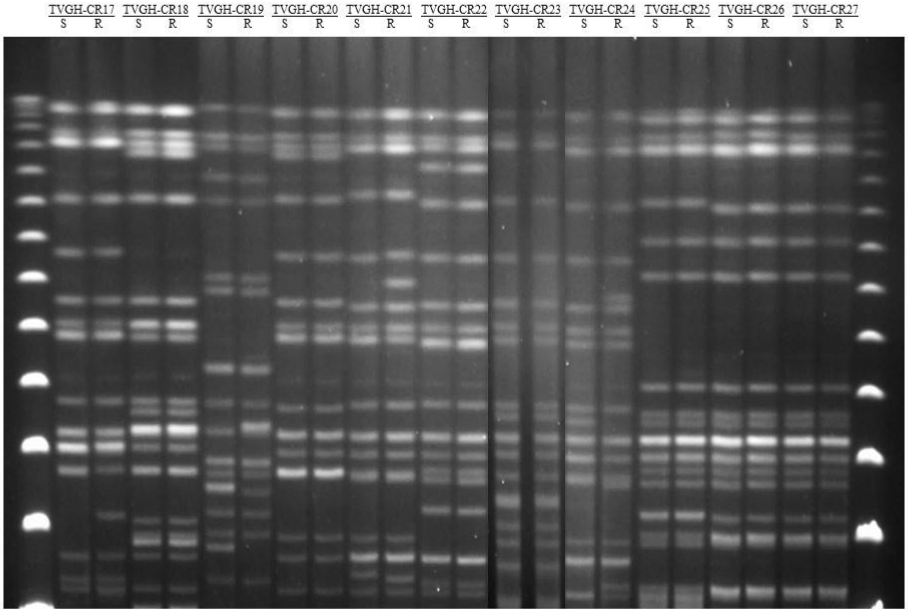

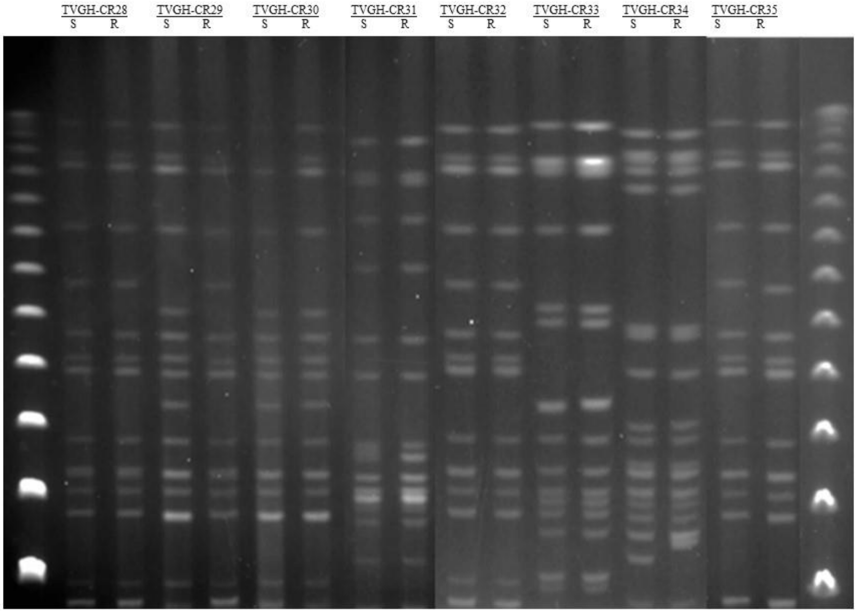

Pulsed-field gel electrophoresis of XbaI-digested genomic DNA was performed for 35 pairs of colistin-resistant and colistin-susceptible carbapenem-resistant *Klebsiella pneumoniae* strains, with ≤ 3 different fragments observed in 30/35 (86%) paired strains.
